# Supplementary material for: Decomposed interaction testing improves detection of genetic modifiers of the relationship of dietary omega-3 fatty acid intake and its plasma biomarkers with hsCRP in the UK Biobank
Source: medRxiv. 2024 Sep 10:2024.09.09.24313018. Preprint. [Version 1] doi: 10.1101/2024.09.09.24313018 (PMC11419197; doi:10.1101/2024.09.09.24313018)
Supplement: Supplement 2 [file NIHPP2024.09.09.24313018v1-supplement-2.pdf]

medRxiv preprint doi: https://doi.org/10.1101/2024.09.09.24313018; this version posted September 10, 2024. The copyright holder for this preprint (which was not certified by peer review) is the author/funder, who has granted medRxiv a license to display the preprint in perpetuity.

It is made available under a CC-BY 4.0 International license .

Supplementary Figures

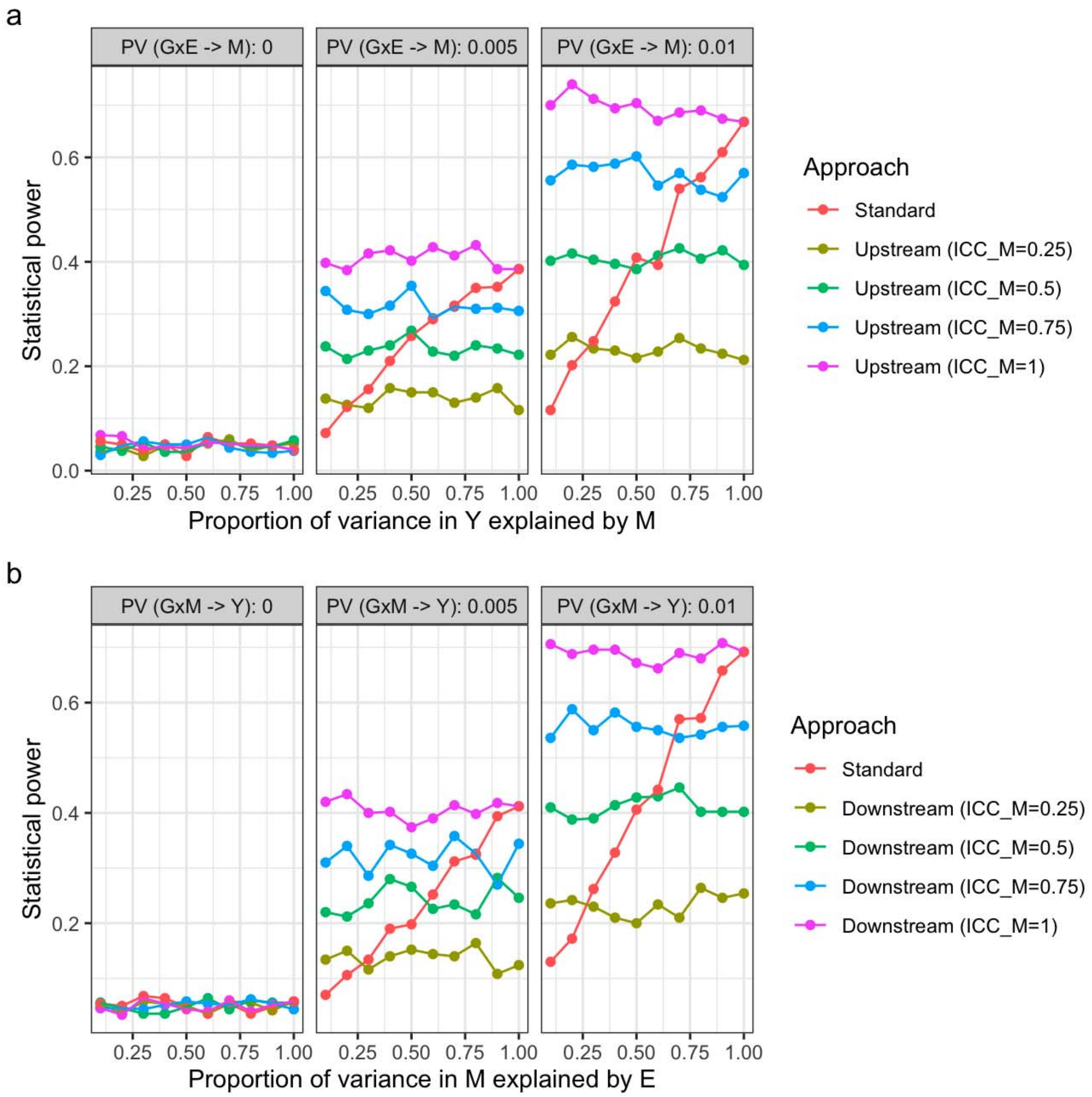

Supplementary Figure S1: Statistical power simulation results for the decomposed versus standard approaches. *X*-axes correspond to the strength (quantified by proportion of variance explained) of the M-Y relationship (upstream, panel a) or E-M relationship (downstream, panel b). Faceted panels correspond to the strength of the simulated genetic interaction with E (upstream) or M (downstream). Colors correspond to the choice of test and associated measurement error in M (quantified by the ICC, where 0 indicates no signal and 1 indicates perfect measurement).

medRxiv preprint doi: https://doi.org/10.1101/2024.09.09.24313018; this version posted September 10, 2024. The copyright holder for this preprint (which was not certified by peer review) is the author/funder, who has granted medRxiv a license to display the preprint in perpetuity.

It is made available under a CC-BY 4.0 International license .

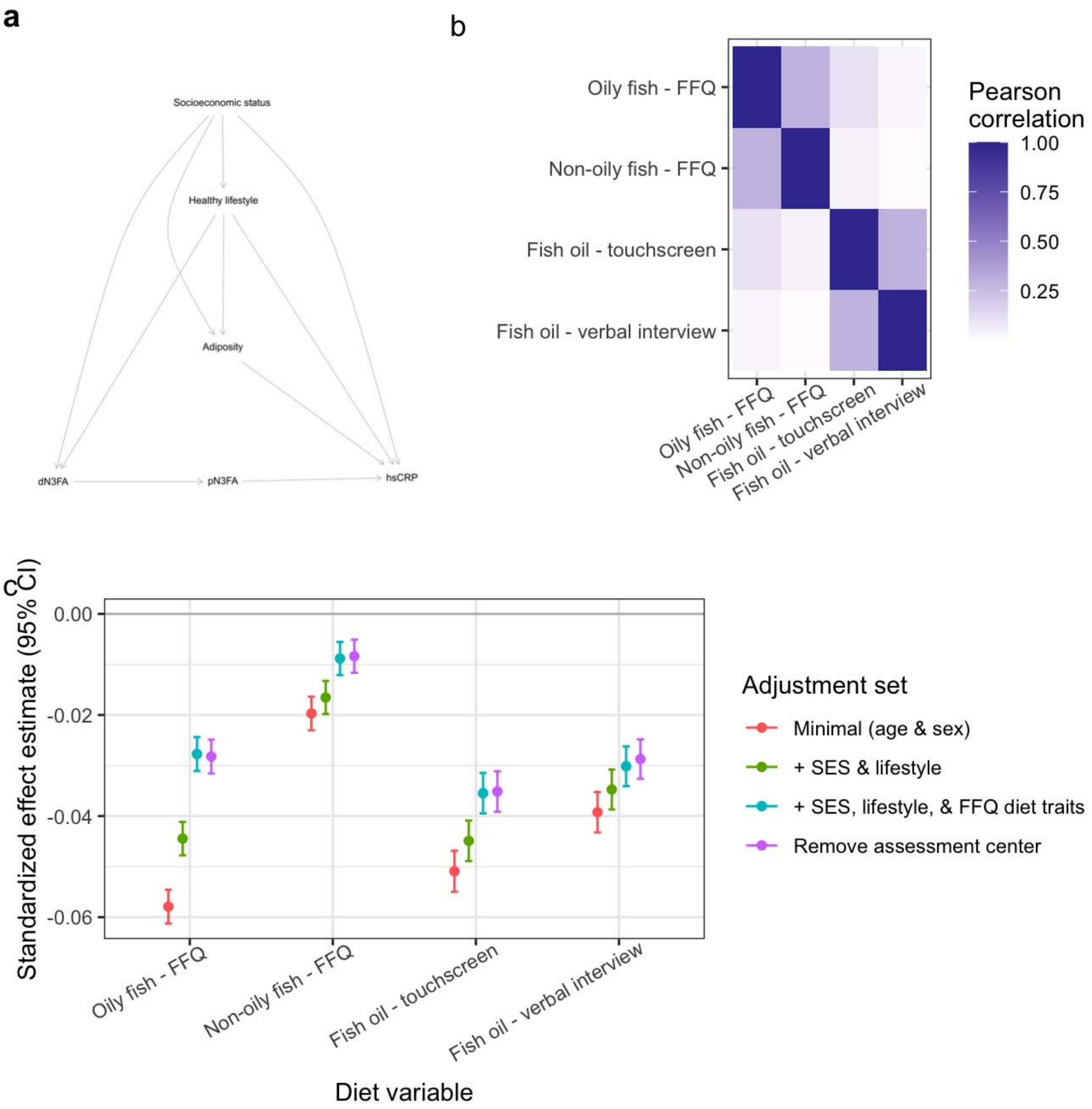

Supplementary Figure S2: N3FA exposure and covariate selection. a) Directed acyclic graph informing covariate selection based on dN3FA-hsCRP main effect, with mediation by pN3FA. b) Heatmap of correlations between key N3FA-containing dietary variables (fish) and supplements (fish oil). c) Effect estimates for the relationship between these variables and log-transformed hsCRP (standardized to units of $SD_{outcome}/SD_{exposure}$). Colors correspond to confounder adjustment sets (SES = socioeconomic status indicators; purple points correspond to estimates adjusted for the full set of covariates minus all categorical indicators for assessment center; see Methods).

medRxiv preprint doi: https://doi.org/10.1101/2024.09.09.24313018; this version posted September 10, 2024. The copyright holder for this preprint (which was not certified by peer review) is the author/funder, who has granted medRxiv a license to display the preprint in perpetuity.

It is made available under a CC-BY 4.0 International license .

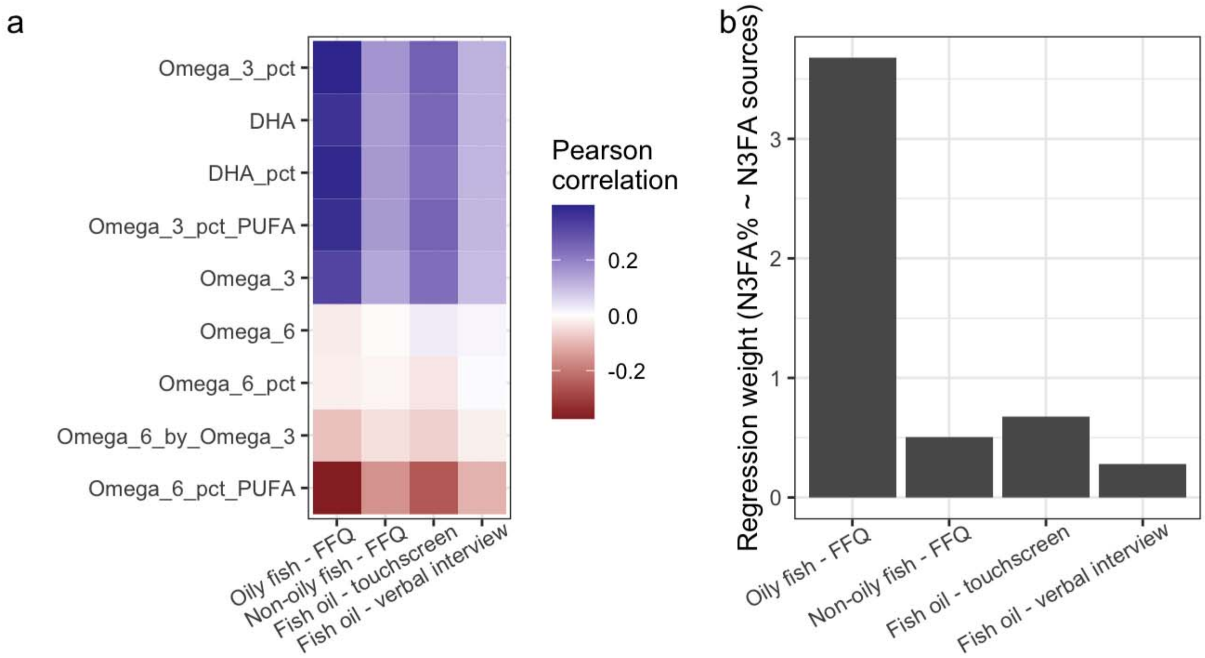

Supplementary Figure S3: Mediator selection and imputation of dietary N3FA (dN3FA). a) Heatmap of correlations between fish and fish oil variables and various omega-3 and omega-6 species available in the Nightingale metabolomics data. b) Multivariable regression effect estimates for each of these dietary variables on pN3FA (plasma omega-3 as a percentage of total fatty acids). Units for exposures are servings/day for fish variables and binary yes/no indicators for fish oil variables.

medRxiv preprint doi: https://doi.org/10.1101/2024.09.09.24313018; this version posted September 10, 2024. The copyright holder for this preprint (which was not certified by peer review) is the author/funder, who has granted medRxiv a license to display the preprint in perpetuity.
It is made available under a CC-BY 4.0 International license .

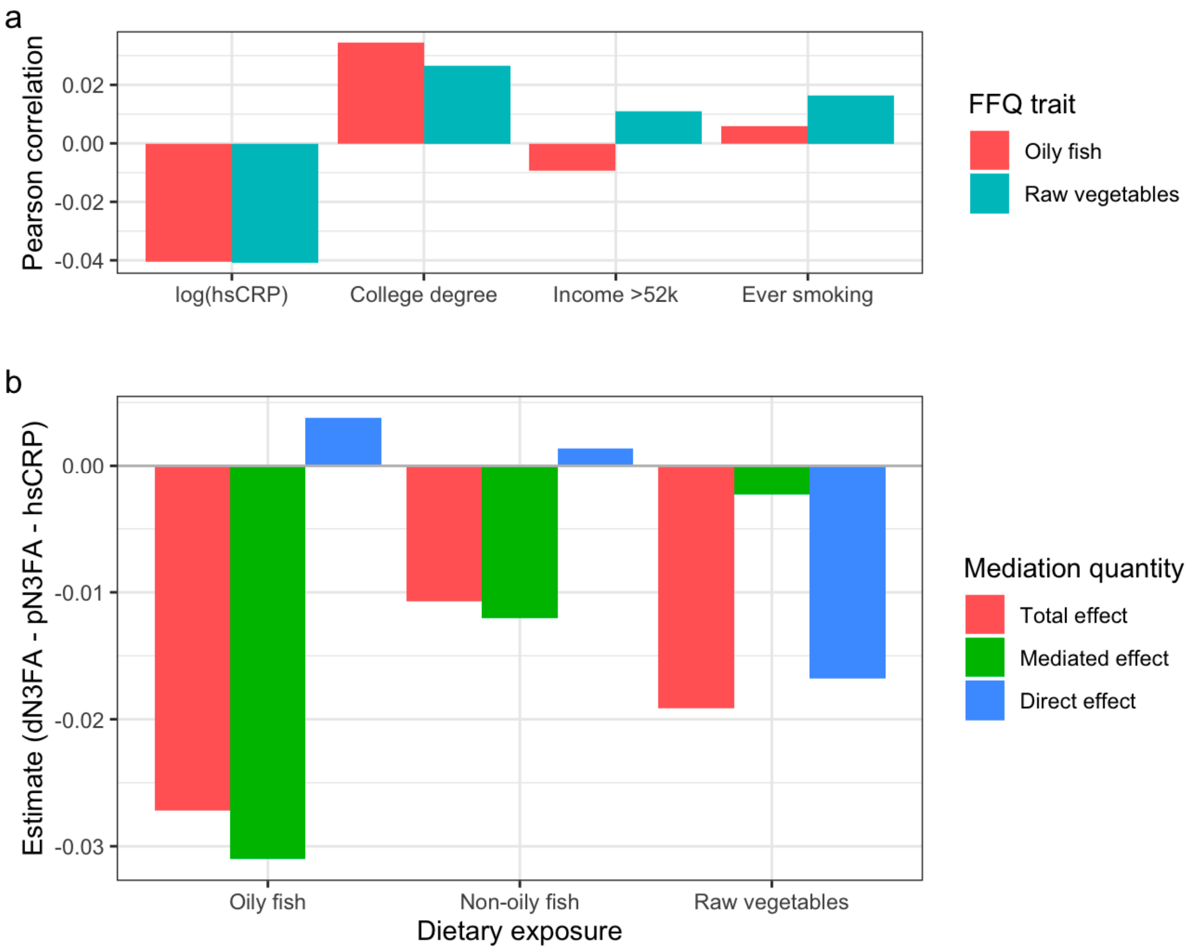

Supplementary Figure S4: Raw vegetable intake as a negative control for mediation results. a) Barplot displays Pearson correlations between oily fish (variable of interest) and raw vegetable (negative control) intake and other variables of interest. b) Mediation testing results for the impact of oily fish (a primary N3FA source), non-oily fish (a modest N3FA source), and raw vegetable intake on log(hsCRP) through pN3FA as a mediator. Colors correspond to various estimates derived from mediation testing Monte Carlo draws (see Methods).

medRxiv preprint doi: https://doi.org/10.1101/2024.09.09.24313018; this version posted September 10, 2024. The copyright holder for this preprint (which was not certified by peer review) is the author/funder, who has granted medRxiv a license to display the preprint in perpetuity.

It is made available under a CC-BY 4.0 International license .

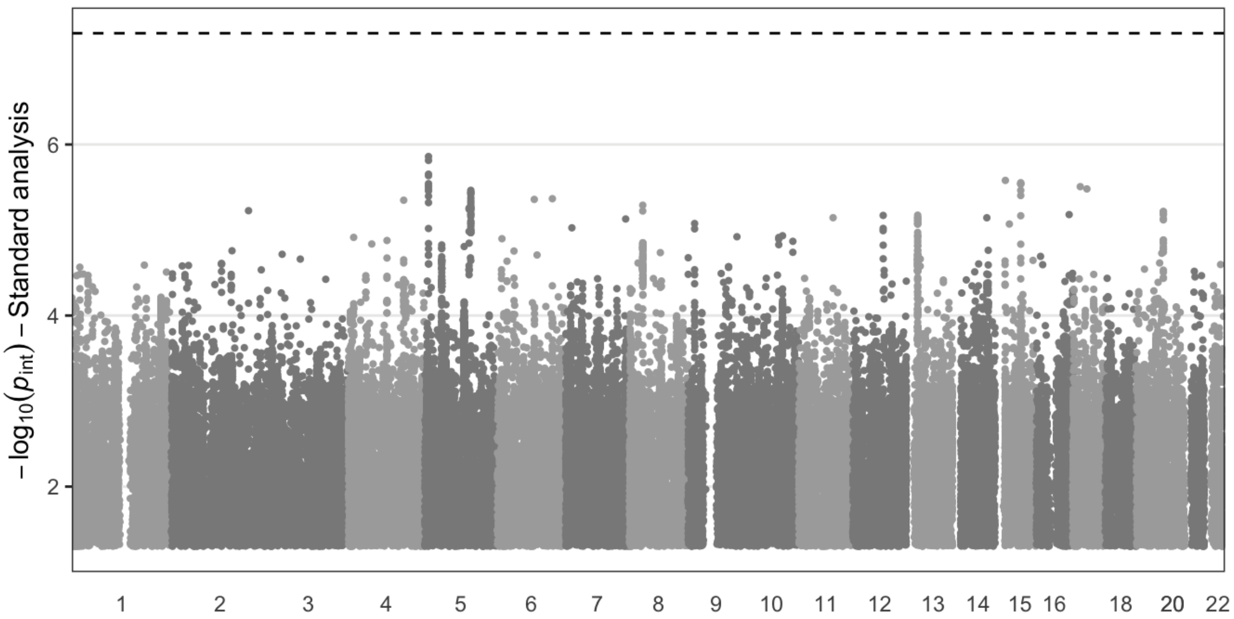

Supplementary Figure S5: Manhattan plot displays variant interaction *p*-values as a function of chromosomal position for the standard analysis approach (dN3FA exposure and hsCRP outcome). The *y*-axis shows -log(*p*) for interaction tests (based on robust standard errors), while the *x*-axis indicates chromosomal position. Dotted line indicates the genome-wide significance threshold of 5×10^{-8}.
